# A Multidisciplinary, Open Access Platform for Research on Biomolecules

**DOI:** 10.3390/biom1010001

**Published:** 2011-08-22

**Authors:** Jürg Bähler

**Affiliations:** Department of Genetics, Evolution & Environment and UCL Cancer Institute, University College London, Darwin Building, Gower Street, London WC1E 6BT, UK; E-Mail: j.bahler@ucl.ac.uk; Tel: +44(0)203 108 1602; Fax: +44(0)20 7679 7096

I am pleased to introduce *Biomolecules*, a new journal to report on all aspects of science that focuses on biologically derived substances, from small molecules to complex polymers. Some examples are lipids, carbohydrates, vitamins, hormones, amino acids, nucleotides, peptides, RNA and polysaccharides, but this list is far from exhaustive. Research on biomolecules encompasses multiple fascinating questions. How are biomolecules synthesized and modified? What are their structures and interactions with other biomolecules? How do biomolecules function in biological processes, at the level of organelles, cells, organs, organisms, or even ecosystems? How do biomolecules affect either the organism that produces them or other organisms of the same or different species? How are biomolecules shaped by evolution, and how in turn do they affect cellular phenotypes? What is the systems-level contribution of biomolecules to biological function?

The scope of *Biomolecules* is broad and multidisciplinary, covering biochemical, molecular, cell biological, genetic, physiological, and computational approaches to name a few. I anticipate that this journal will foster fruitful crosstalk between the various disciplines and approaches applied to biomolecule research. We will publish any manuscript of high scientific quality that pertains to diverse aspects relevant to biogenic substances, irrespective of biological question or methodology.

To kick-start *Biomolecules*, we will also publish ambitious series of special issues that cover selected topics of current interest and relevance, including both reviews and original research. Example topics are non-coding RNAs, DNA damage responses, protein folding, sumoylation, and glycoproteins. We welcome suggestions for additional topics. These special issues will be edited by respected leaders in the specific fields to increase the profile and visibility of the papers.

The open access format of *Biomolecules* will provide effective and unrestricted dissemination of the papers to a wide readership. This format will help to realize the ambition of the journal to promote stimulating research for readers with multiple backgrounds and perspectives. We aim at competent, fair peer review and rapid publication to make it attractive for prospective authors to submit to *Biomolecules*.

On behalf of the editorial office and board, I welcome all authors and reviewers and thank them for all their valuable contributions to this exciting new journal. Together we can develop *Biomolecules* into a respected venue for the fast and cost-effective publication of quality research from diverse scientists across the globe. We look very much forward to working with you all.

